# Prediction of Response to Treatment in Children with Epilepsy

**Published:** 2018

**Authors:** Mohammad GHOFRANI, Mohammad Mehdi NASEHI, Sasan SAKET, Mohsen MOLLAMOHAMMADI, Mohammad Mahdi TAGHDIRI, Parvaneh KARIMZADEH, Seyed Hassan TONEKABONI, Mohsen JAVADZADEH, Narjes JAFARI, Azadeh ZAVEHZAD, Masoud HASANVAND AMOUZADEH, Mahsa BESHRAT, Meysam BABAEI

**Affiliations:** 1 Child Neurology Research Center, Shahid Beheshti University of Medical Sciences, Tehran, Iran; 2 Department of Pediatric Neurology, Qom University of Medical Sciences, Qom, Iran; 3 Department of Pediatric, Faculty of Medicine, Kashan University of Medical Sciences, Kashan, Iran.

**Keywords:** Pediatric, Anti-seizure drug, Response to treatment, Children, Epilepsy

## Abstract

**Objective:**

This study was conducted to predict the response to treatment in patients treated with anti-epilepsy drugs.

**Material and Methods:**

This analytical questionnaire-based study was conducted in 2014 among 128 patients with epilepsy admitted to Mofid Children's Hospital, Tehran, Iran. The inclusion criteria were children 2 months to 12 yr of age with epilepsy and patients who experienced fever and seizure attacks at least once were excluded from the study. Patients were followed up for 6 months and the response to their treatment was recorded. The good response to treatment was defined as the absence of seizure with two drugs during follow up.

**Results:**

Seventy-two patients (56.3%) were boys. The age of the first seizure was under 2 yr old in 90 patients (70.3%). History of febrile convulsion, family history of epilepsy and history of asphyxia was found in 16 (12.5%), 41 (32%), and 27 (21.1%) patients, respectively. Seizure etiology was idiopathic in 90 patients (70.3%), and the number of seizures was 1-2 in 36 patients (28.1%). Overall, 57 patients (44.5%) had cerebral lesion according to CT scan or MRI, and EEG was abnormal in 101 patients (78.9%). In 6-month follow-up, 40 patients (31.3%) responded well to the treatment and 88 patients (68.8%) responded poorly to the treatment. History of asphyxia (OR = 6.82), neonatal jaundice (OR = 2.81) and abnormal EEG (OR = 0.19) were effective factors in response to treatment.

**Conclusion:**

Abnormal EEG is an effective factor in treatment response in the children studied.

## Introduction

Seizure is a chronic neurological disease, defined as epilepsy when it occurs 2 times or more (1). “The cumulative incidence of epilepsy is 3% in a lifetime, with more than half of the cases occurring in childhood. However, the annual incidence of epilepsy is 0.5%-0.8%” (2). A high prevalence of seizure was in Iranian society and among children under 12 yr old in Tehran, as 32 per 1000 people (3). 

Less than half of seizures in childhood are epileptic seizures and in which recurrent seizures are stimulated in the brain. Epilepsy improves in many children. A seizure can be the sign of a serious underlying disorder in the central nervous system or a systemic disease that requires full-fledged examination and treatment. The prognosis of children with epilepsy is usually good (2), but in 10% to 20% of children with seizure, the attacks still continue despite appropriate treatment (4). “After the first provoked seizure in children, the risk of relapse is about 50% and after the second seizure, the risk is about 80%” (5). 

The biological basis of seizure relapse is probably multifactorial and may include severe syndromes or underlying neuropathological diseases, abnormal reorganization of neurons, replacement in receptors and neurotransmitters, ion channel abnormalities, reactive autoimmunity and inappropriate use of antiepileptic drugs (6). After the first seizure, if the patient is normal in terms of neuronal development according to EEG and MRI tests, the risk of relapse is low and usually, the patient does not need to start treatment. However, if the patient's EEG and MRI, neurodevelopment and neurological examination show abnormalities and they have positive family history of epilepsy, the risk of relapse is high and treatment should be started immediately (7). The determining factors of response to treatment in patients with epilepsy include etiology of epilepsy, epilepsy type, underlying syndrome and the frequency seizure. Environmental factors such as trauma and the simultaneous use of drugs and genetic factors in the metabolism of drugs may play a role in the therapeutic response (2). About 60%-70% of children with epilepsy who become seizure-free can successfully stop their treatment for 1-2 year (8, 9). Therefore, predicting the response to treatment in these patients is always a major challenge. 

In previous studies, the age of first seizure, intellectual power status and neonatal seizure were considered as predictive factors (10, 11). Therefore, this study was conducted to predict the response to treatment in patients with epilepsy based on the factors that affect the disease and based on other factors that are prevalent in Iran and can influence the prognosis.

## Materials and Methods

This analytical questionnaire-based study was conducted in 2014 among patients with epilepsy admitted to Mofid Children's Hospital, Tehran, Iran as a level three referral hospital in Tehran. The inclusion criteria were children 2 months to 12 yr of age with epilepsy and patients who experienced febrile seizure attacks at least once were excluded from the study. Samples were selected using improbable sampling and convenience sampling techniques. 

The obtained sample size was 128 people considering the main outcome of the study, which was proper response to therapy at the end of the sixth month and based on 66% response, significance level of 0.05, 8% accuracy and the corresponding formula. Epilepsy was diagnosed by a pediatric neurologist. After the initial recording of patient information, patients were followed up for 6 months and the response to their treatment was recorded. The studied variables were as follows: Response to treatment, age of first seizure, gender, family history of seizure, history of birth asphyxia, history of neonatal seizure, IQ, the frequency of seizure before treatment, etiology, seizure type, sign of lesion in CT scan or MRI and EEG status. The good response to treatment was defined as the absence of maximum seizure with two drugs during follow up. 

Data were analyzed using SPSS (ver.20, Chicago, IL, USA) and logistic regression model was used to determine the predictive power of each of the variables studied in the prognosis of the disease. *P*<0.05 was considered as significance level.

## Results

The present study was conducted among 128 children with seizure treated with antiepileptic drugs. Overall, 72 patients (56.3%) were boys. The age of the first seizure was under 2 yr old in 90 patients (70.3%). Overall, 108 patients (84.4%) lived in the city, and the type of delivery was vaginal for 63 patients (49.2%). History of febrile seizure was found in 16 patients (12.5%), family history of seizure was found in 41 patients (32%), history of asphyxia in 27 (21.1%), history of neonatal seizure in 24 patients (18.8%) and neonatal jaundice in 38 patients (29.7%). IQ was was decreased in 79 patients (61.7%). Seizure etiology was idiopathic in 90 patients (70.3%), and the number of seizures was 3-20 in 51 patients (39.8%). Fifty-seven patients (44.5%) had cerebral lesion according to CT scan or MRI, and EEG was abnormal in 101 patients (78.9%). 

The type of seizure was generalized tonic-clonic in 35 patients (27.3%) , simple partial in 13 patients (10.2%), infant spasm in 5 patients (3.9%), tonic in 16 patients (12.5%), atonic in 5 patients (3.9%), myoclonic in 16 patients (12.5%), mixed in 14 patients (10.9%), complex partial in 4 patients (3.1%), 1 case of absence (0.8%) and 1 case clonic (0.8%). 

In 3-months follow-up, 43 patients (33.6%) responded well to the treatment, 75 patients (58.6%) responded poorly to the treatment and 10 patients (7.8%) had no response to treatment. In 6-month follow-up, 40 patients (31.3%) responded well to the treatment and 88 patients (68.8%) responded poorly to the treatment. Mortality was not observed in any of the patients. 

Table 1 presents the distribution of effective factors in response to treatment according to the status of response to treatment, 6 months after starting the treatment. History of asphyxia (OR = 6.82), neonatal jaundice (OR = 2.81) and abnormal EEG (OR = 0.19) were effective factors in response to treatment (Table 2). 

**Table 1:** The distribution of effective factors in response to treatment after 6 months

		Good (n=40) N (%)	Poor (n=88) N (%)	*P*-value
Age at first seizure	<2 yr	25 (62.5)	65 (73.9)	0.07
	2-5 yr	6 (15)	16 (18.2)	
	>6 yr	9 (22.5)	7 (8)	
Sex	Female	16 (40)	40 (45.5)	0.701
	Male	24 (60)	48 (54.5)	
Delivery	NVD	19 (47.5)	44 (50)	0.85
	Cesarian	21 (52.5)	44 (50)	
History of febrile convulsions		4 (10)	12 (13.6)	0.774
Familial history of epilepsy		13 (32.5)	28 (31.8)	1
Asphyxia		14 (35)	13 (14.8)	0.018
Neonatal seizure		6 (15)	18 (20.5)	0.626
intelligence		18 (45)	61 (69.3)	0.011
Number of seizures	1-2	11 (27.5)	25 (28.4)	0.994
	3-20	16 (40)	35 (39.8)	
	>20	13 (32.5)	28 (31.8)	
Neonatal Icter		18 (45)	20 (22.7)	0.013
Etiology	Idiopathic	32 (80)	58 (65.9)	0.144
	Symptomatic	8 (20)	30 (34.1)	
Abnormal EEG		27 (69.2)	74 (89.2)	0.01
Abnormal MRI		11 (25.7)	46 (52.3)	0.012

**Table 2:** The multivariate analysis of effective factors in response to treatment after 6 months

*Variable*		*OR*	*CI95%*	*P-value*
Age at first seizure	<2 yr	-	-	0.197
	2-5 yr	0.270	0.065-1.121	0.071
	>6 yr	0.351	0.065-1.906	0.225
Sex (male)		1.750	0.640-4.788	0.276
Delivery (cesarean section)		0.706	0.262-1.905	0.492
History of febrile convulsions		0.442	0.085-2.286	0.330
Familial history of seizure		0.825	0.283-2.404	0.725
Asyphexia		6.825	1.960-23.767	0.003
Neonatal seizure		0.937	0.202-4.351	0.934
Low IQ		0.479	0.165-1.391	0.176
Number of seizures	1-2	-	-	0.866
	3-20	0.722	0.220-2.372	0.591
	>20	0.860	0.272-2.717	0.798
Icter		2.810	1.019-7.749	0.046
Etiology (idiopathic)		2.268	0.578-8.901	0.241
Abnormal EEG		0.195	0.056-0.676	0.010
Abnormal MRI		0.537	0.171-1.690	0.288

## Discussion

The present study aimed to determine the treatment response of patients with epilepsy based on the factors effective in the disease. Abnormal EEG in both single-variable and multivariate analyses are an effective factor in treatment response, where abnormal EEG was significantly associated with poor treatment response. In a study on 161 children with epilepsy, 23 predictors for treatment response in children were examined. Age at the onset of the disease, type of seizure, abnormal spike-waves in EEG one year after treatment, and presence of 3-Hz spike-wave in patients’ EEG in the first 6 months of treatment were effective factors in treatment response and disease control (11). Children without adverse factors may have 80%-90% success rate because factors have additive effect, and those with all adverse factors may only have a success rate of 10%-20% (5).

In the present study, a relationship was found between IQ and treatment response in the single-variable analysis. Such relationship was not observed in the multivariate analysis. Normal intelligence (or normal rational power), onset of epilepsy in the age less than 6 yr, lack of history of epilepticus status, and lack of seizure in the first week of treatment were among the predictors for controlling seizure (10). In Bangladeshi epileptic children at the age of 2 months to 15 yr, motor disorder, cognitive impairment, and multiple seizure types were reported to be effective clinical factors in predicting epilepsy control and treatment response (12).

In the present study, history of febrile convulsion and history of seizure in the neonatal period did not correlate with response to treatment. About 5%-15% of cases of epilepsy in children evolve to resistant epilepsies. Factors affecting the resistance of epilepsy are symptomatic causes including focal epilepsies with an early unfavorable course, Landau-Kleffner epilepsy or continuous spike or wave during sleep (CSWS), cognitive impairment syndromes, and syndromes similar to West Lennox Gastaut or Dravet's syndromes (13).

A relationship was found between lesion in the CT scan or MRI with the treatment response in the single-variable analysis but not in the multivariate analysis. A comparative study of prognostic factors was conducted between two groups of patients who responded to treatment and those who did not respond to treatment and found that the number of cases with abnormal brain imaging results was significantly higher in the group who did not respond to treatment (14). 

A limitation of the present study was the small sample size and a limited follow-up period.

**In conclusion,** results of univariate and multivariate analysis indicated that abnormal EEG is an effective factor in treatment response in children.
